# *Staphylococcus* spp. Isolated from Bovine Subclinical Mastitis in Different Regions of Brazil: Molecular Typing and Biofilm Gene Expression Analysis by RT-qPCR

**DOI:** 10.3390/antibiotics9120888

**Published:** 2020-12-10

**Authors:** Priscila Luiza Mello, Danilo Flávio Moraes Riboli, Lisiane de Almeida Martins, Maria Aparecida Vasconcelos Paiva Brito, Cassiano Victória, Letícia Calixto Romero, Maria de Lourdes Ribeiro de Souza da Cunha

**Affiliations:** 1 Departamento de Ciências Químicas e Biológicas, Setor Microbiologia e Imunologia, Instituto de Biociências, Universidade Estadual Paulista—UNESP, Botucatu, SP 18618-689, Brazil; priscila.mello@prof.ung.br (P.L.M.); danilo.riboli@unesp.br (D.F.M.R.); leticia.calixto@unesp.br (L.C.R.); 2 Docente no Programa de Pós Graduação em Enfermagem, Universidade Guarulhos—UNG, Guarulhos, SP 07023-070, Brazil; 3 Universidade Paranaense—UNIPAR, Umuarama, PR 87502-210, Brazil; lisiane.almeida.martins@gmail.com; 4 Empresa Brasileira de Pesquisa Agropecuária—EMBRAPA, Juiz de Fora, MG 36038-330, Brazil; mavpaiva@gmail.com; 5 Departamento de Higiene Veterinária e Saúde Pública da Faculdade de Medicina Veterinária e Zootecnia, Universidade Estadual Paulista—UNESP, Botucatu, SP 18618-681, Brazil; cassiano.victoria@unesp.br

**Keywords:** CoNS, *Staphylococcusaureus*, biofilm, gene expression, molecular epidemiology

## Abstract

Bovine mastitis is mainly caused by bacteria of the genus *Staphylococcus* spp., which possess different virulence factors, including the capacity for biofilm formation that provides enhanced protection against the action of immune system components and serves as a barrier against the penetration of antimicrobial agents. This study aimed to characterize 181 *Staphylococcus* spp. Strains—including *Staphylococcus* *aureus* and coagulase-negative staphylococci (CoNS) isolated from bovine subclinical mastitis in six Brazilian states—by molecular methods. RT-qPCR was used to verify the expression of genes of the *ica* operon—mainly responsible for biofilm formation—as well as *bap* and *bhp*. Chromosome similarity among the isolates was investigated by pulsed-field gel electrophoresis (PFGE) and multilocus sequence typing (MLST). The *ica*A gene was detected in 79 (43.6%) isolates, *ica*B in 24 (13.2%), *ica*C in 57 (31.4%), and *ica*D in 127 (70.1%). The *bap* gene was identified in 66 (36.4%) isolates, while the *bhp* gene was found in nine (4.9%). RT-qPCR confirmed the expression of the *ica*A gene in 60 (75.9%) isolates, of *ica*B in six (25%), of *ica*C in 26 (45.6%), and of *ica*D in 80 (63%). Clonal typing of the isolates by PFGE permitted the identification of eight *Staphylococcus* *aureus* clusters that simultaneously included ≥3 strains, with a similarity of ≥80%. Regarding the other species studied, three clusters were observed for *Staphylococcus* *chromogenes* and four clusters for *Staphylococcus* *epidermidis*. Only one cluster each was identified for *Staphylococcus* *saprophyticus* and *Staphylococcus* *simulans*, while the other species did not form any cluster. With respect to MLST, ST126 and ST1 were the prevalent sequence types in *S. aureus*, while in *S.* *epidermidis* all sequence types were different. These results reveal strains with the same evolutionary origin as other isolates, which might cause infections in humans and animals, suggesting their ability to spread between these species.

## 1. Introduction

Inadequate handling of livestock can trigger inflammatory processes in the mammary gland, known as mastitis, the most prevalent and costly disease in the dairy industry. Mastitis is caused by different microorganisms that directly influence the physicochemical characteristics, composition and cellularity of milk [1,2]. The economic consequences of both clinical and subclinical mastitis include losses due to the costs of treatment, lower milk production, changes in product quality, and culling [2]. Besides the economic losses, mastitis is a public health hazard since it can cause zoonoses and food poisoning [3,4].

Brazil is the world’s fourth-largest milk producer and the south is the main production region, responsible for 35% of the milk produced and for 30.6% of the total number of herds in the country (Table S1), followed by the southeast region [5,6]. Few studies report the prevalence of subclinical mastitis in Brazilian dairy herds [5]. At any rate, wide variation exists in the cow-level prevalence of this disease.

Epidemiological studies have demonstrated the presence of the genus *Staphylococcus* in approximately 50% of cases of bovine mastitis [7]. Bovine mastitis can be caused by different microorganisms and bacteria of the genus *Staphylococcus* are the most common [7,8]. *Staphylococcus aureus* is the main etiological agent of contagious bovine mastitis. However, coagulase-negative staphylococci (CoNS) are a group of microorganisms that are increasingly being isolated from bovine subclinical intramammary infections in several countries [8,9,10]. Strains of *Staphylococcus chromogenes*, *Staphylococcus simulans, Staphylococcus haemolyticus, Staphylococcus xylosus* and *Staphylococcus epidermidis* can persist in the udder, causing a low to moderate increase in somatic cell count and possibly a slight reduction of milk production [11]. Despite the common isolation of these species from secretions of the bovine mammary glands, little is known about the molecular characteristics of CoNS [12].

*Staphylococcus* species possess different virulence factors, including the capacity for biofilm formation [13]. The biofilm protects the bacteria against the action of immune system components by blocking phagocyte activity [14], and serves as a barrier that impairs the penetration of antimicrobial agents [15]. Moreover, chronic bacterial infections are often attributable to biofilms due to their high tolerance to conventional antibiotic therapies [16]. Fox et al. (2005) drew attention to the role of biofilms as a selective advantage in the pathogenesis of mastitis, which might be due to a strain characteristic or genetically linked to traits [14].

The proliferation of cells to adhere and form a biofilm is mediated by the production of polysaccharide intercellular adhesin (PIA). This adhesin is encoded by the gene product of the *ica* locus of the *ica*ADBC operon, which is essential for biofilm formation and virulence of the microorganisms [17]. Another important gene that also regulates biofilm formation is biofilm-associated protein (*bap*), which encodes the bap surface protein. Unlike PIA which only seems to be involved in intercellular adhesion [18], this protein promotes primary binding to abiotic surfaces and intercellular adhesion. In addition to *bap*, the *bhp* gene is also related to biofilm formation irrespective of the presence of the *ica* operon. The presence and expression of biofilm genes can be analyzed by RT-qPCR, which permits to quantify the genes expressed.

A variety of molecular techniques based on chromosome similarity have been developed over the last decades, including pulsed-field gel electrophoresis (PFGE) and multilocus sequence typing (MLST) [19,20]. PFGE has become an important method and is considered the gold standard for the molecular typing and characterization of bacterial clusters, detecting genetic variations between phylogenetically and epidemiologically related isolates [21]. MLST has been proposed by Maiden et al. (1998) for molecular characterization and epidemiological investigation and has been widely used for phylogenetic interference of bacterial species. The set of alleles at the different loci studied by MLST determines the allelic profile or sequence type (ST) of a bacterial strain. The STs found within a population permit the comparison of its strains, as well as phylogenetic inference of its members [20].

Molecular typing is essential to determine the source of transmission and to study the evolution and molecular organization of these bacteria. Genotyping permits to distinguish between clonal or horizontal dissemination of resistance and is used to monitor the global distribution of resistant bacteria [22]. Within this context, the objective of the present study was to molecularly characterize *S. aureus* and CoNS isolated from bovine subclinical mastitis cases in herds from different Brazilian regions and states, and to analyze biofilm formation by qRT-PCR in order to increase our understanding of these isolates. The findings will contribute to establishing measures to prevent and control the dissemination of pathogenic *Staphylococcus* spp. clusters in Brazil.

## 2. Results

### 2.1. Detection of Genes Involved in Biofilm Formation

The presence of biofilm formation-related genes was investigated in the genomic DNA of all 181 isolates of the study. The *ica*A gene was detected in 79 (43.6%) isolates, *ica*B in 24 (13.2%), *ica*C in 57 (31.4%), and *ica*D in 127 (70.1%). The *bap* gene was detected in 66 (36.4%) isolates, while the *bhp* gene was found in nine (4.9%).

### 2.2. Expression Analysis of Genes of the Ica ADBC Operon by RT-qPCR

**Figure 1:**
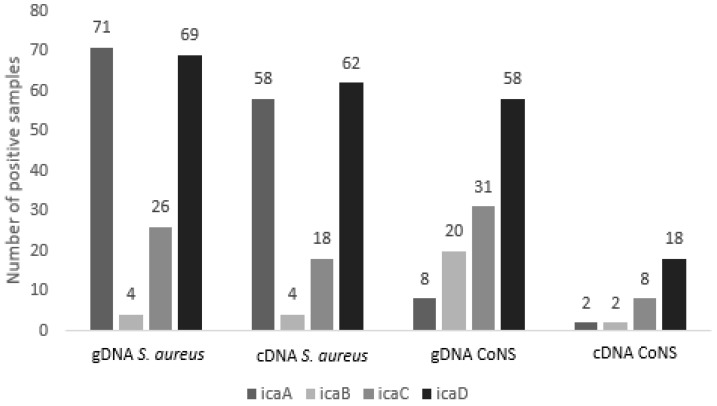
Comparison of the detection of genes of the *ica*ADBC operon by conventional PCR (genomic DNA, gDNA) and expression analysis by RT-qPCR (complementary DNA, cDNA).

Based on the results of genomic DNA analysis, the expression of these genes was analyzed by RT-qPCR using cDNA of the isolates as a template. The *ica*A gene was expressed in 60 (75.9%) isolates, *ica*B was expressed in six (25%), *ica*C was expressed in 26 (45.6%), and *ica*D was expressed in 80 (63%). Figure 1 shows the comparison of detection in genomic DNA and cDNA expression by RT-qPCR. The distribution of the expressed genes according to *Staphylococcus* spp. is shown in Table 1.

Among the 82 *S. aureus* isolates analyzed, the most expressed genes were *icaA* in 58 (70.7%) isolates and *icaD* in 62 (75.6%). Fifty-two isolates (63.4%) simultaneously expressed the two genes. The least expressed gene was *icaB*, which was only expressed in four isolates, while *icaC* was found in 18 isolates. The frequency of genes of the *icaA*DBC operon was lower in CoNS. Six *S. chromogenes* isolates expressed some of the biofilm genes and the expression was also observed in seven *S. epidermidis* isolates.

### 2.3. Interpretation of Susceptibility to Oxacillin and Vancomycin by the E-Test

Interpretation of the E-test results according to the breakpoints defined by the Clinical and Laboratory Standards Institute (CLSI) [23] for determination of the minimum inhibitory concentration (MIC) of oxacillin revealed that 99% of the *S. aureus* isolates were susceptible to this antibiotic, while 32% of the CoNS isolates were resistant, with MIC_50_ of 0.25 µg/mL and MIC_90_ of 1.5 µg/mL for the two groups (Table 2). Analysis of the vancomycin MIC revealed MIC_50_ and MIC_90_ of 0.50 and 1.5 µg/mL, respectively, for *S. aureus* and 1.0 and 1.5 µg/mL for CoNS (Table 2). All *Staphylococcus* spp. isolates analyzed were susceptible to vancomycin.

### 2.4. Pulsed-Field Gel Electrophoresis

**Figure 2:**
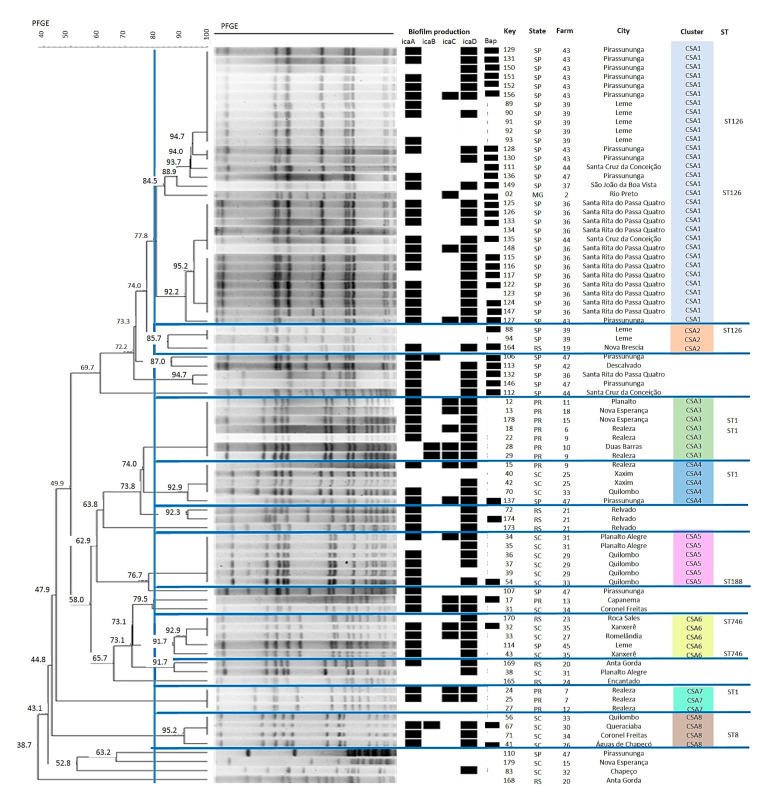
Dendrogram generated from the pulsed-field gel electrophoresis PFGE pattern of *Staphylococcus aureus* isolates (Dice similarity coefficient; clustering method: Unweighted Pair Group Method with Arithmetic Mean- UPGMA).

Molecular typing by PFGE permitted the identification of eight clusters of *S. aureus* isolates that simultaneously included ≥3 strains, with a similarity of ≥80% (Figure 2). A similarity coefficient of 80% was selected to define the pulsed-field type (PFT) clusters after reviewing the epidemiological data associated with each of the clusters of isolates [24]. The largest cluster *Staphylococcus aureus* (CSA1) comprises 31 isolates from São Paulo (SP) or Minas Gerais (MG). This cluster includes isolates from seven different farms, indicating dissemination of this cluster in the region studied. This heterogeneity in the origin of strains was also observed for the other *S. aureus* clusters.

**Figure 3:**
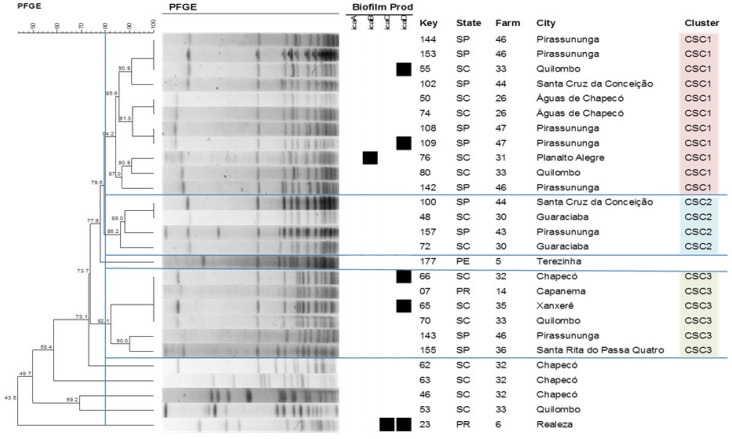
Dendrogram generated from the PFGE pattern of *Staphylococcus chromogenes* isolates (Dice similarity coefficient; clustering method: UPGMA). All *Staphylococcus chromogenes* isolates were negative for the *bhp* gene.

**Figure 4:**
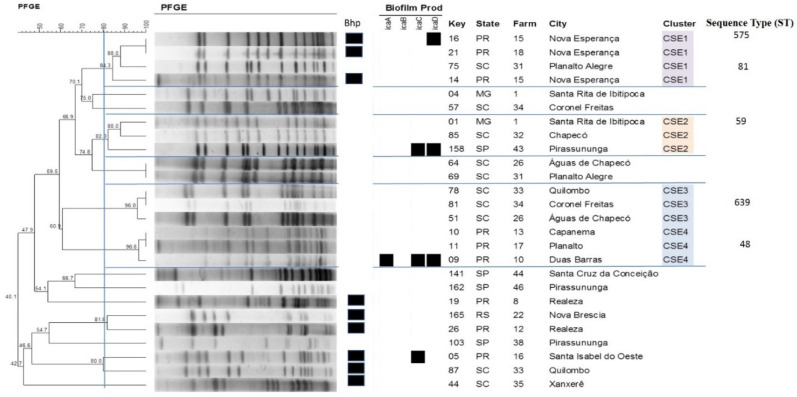
Dendrogram generated from the PFGE pattern of *Staphylococcus epidermidis* isolates (Dice similarity coefficient; clustering method: UPGMA).

**Figure 5:**
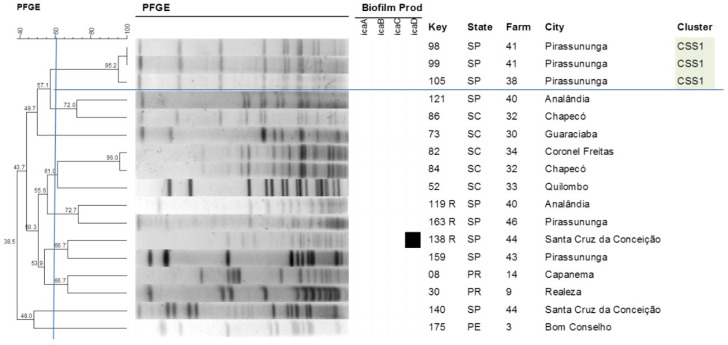
Dendrogram generated from the PFGE pattern of *Staphylococcus saprophyticus* isolates (Dice similarity coefficient; clustering method: UPGMA). All *Staphylococcus saprophyticus* isolates were negative for the *bhp* gene.

**Figure 6:**
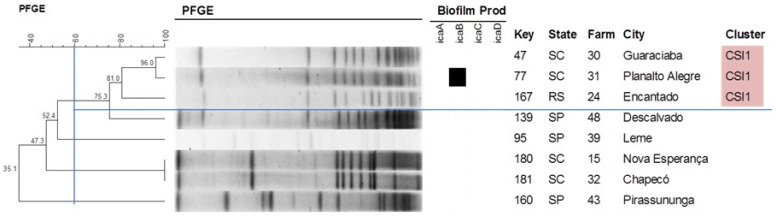
Dendrogram generated from the PFGE pattern of *Staphylococcus simulans* isolates (Dice similarity coefficient; clustering method: UPGMA). All *Staphylococcus simulans* isolates were negative for the *bhp* gene.

With respect to the other species studied, the formation of three clusters was observed for *S. chromogenes* (Figure 3), with cluster *Staphylococcus chromogenes* (CSC1) comprising the largest number of isolates (11) that originated from seven different farms. Four clusters could be identified for *S. epidermidis* (Figure 4), with isolates of the same cluster originating from different states, such as cluster *Staphylococcus epidermidis* (CSE1) that includes isolates from Santa Catarina (SC) and Paraná (PR) and cluster 2 (CSE2) that includes isolates from MG, SC and SP. On the other hand, only one cluster each was observed for *S. saprophyticus* and *S. simulans* (Figure 5 and Figure 6). Table 3 shows the distribution of species according to the electrophoretic pattern, their origin, and biofilm gene expression. The remaining CoNS species did not form clusters according to the criteria adopted.

**Figure 7:**
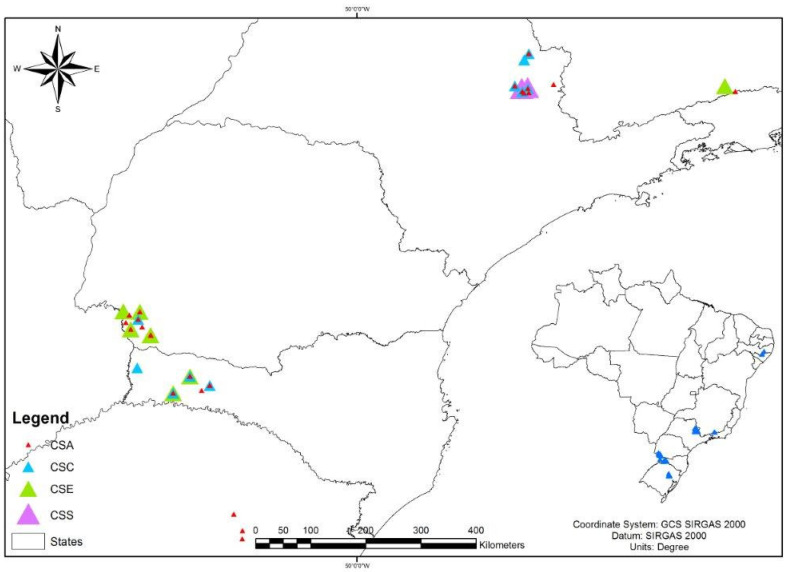
Map with the spatial distribution of the clusters of different *Staphylococcus* species analyzed by PFGE. CSA: cluster *S. aureus*; CSC: cluster *S. chromogenes*; CSE: cluster *S. epidermidis*; CSS: cluster *S. saprophyticus*.

The spatial analysis made it possible to verify the cluster distribution around the area studied (Figure 7).

### 2.5. Multilocus Sequence Typing

A group of 10 *S. aureus* and five *S. epidermidis* isolates were selected for MLST according to the criterion that at least one strain of each cluster described by PFGE would be included.

Six different STs were identified in *S. aureus*. The most prevalent STs were ST126 and ST1, which belonged to CC126 and CC1, respectively. Regarding the geographic distribution of the STs, ST126 (CC126) was detected in isolates from MG and SP, ST1 (CC1) only in isolates from PR, ST746 (CC97) in isolates from RS and SC, and ST8 and ST188 only in isolates from SC.

For *S. epidermidis*, the STs were all different even when the isolates were clustered by PFGE. In the case of one isolate, no exact combination was found for its set of alleles, probably because it is a new ST. The sequence of the combination of alleles was therefore reported to the curator of the pubmlst.org/multilocus-sequence-typing site for the addition of the new ST.

Table 4 and Table 5 show the origin of the isolates, the PFGE cluster to which they belong, and the combination of alleles for *S. aureus* and *S. epidermidis*, respectively. The susceptibility profile of the isolates according to sequence type is given in Table 6.

## 3. Discussion

The genes of the *ica* operon were investigated in the genomic DNA of all isolates studied. A high percentage of these genes was detected in *S. aureus*, in which the *icaD* gene was the most common, followed by *icaA*. Based on these results, gene expression analysis was performed, which confirmed that the *icaD* gene was the most expressed in the isolates of the present study. Fifty-two (63.4%) of the isolates simultaneously expressed the *icaA* and *icaD* genes. Vasudevan et al. (2003), investigating 35 *S. aureus* isolates from bovine mastitis cases, detected biofilm formation in 91.4% of the isolates; 100% were positive for the *icaA* and *icaD* genes [25]. A high frequency of *icaD*-positive isolates has been reported in other studies investigating *S. aureus* from mastitis. In the study of Krewer et al. (2015), 92.8% of the isolates exhibiting adherence to microplates carried the *icaD* gene [26].

Comparison of the frequency of *icaADBC* genes present in CoNS showed a major difference between detection in genomic DNA and cDNA. The presence of a gene does not indicate that it will be expressed. According to Rode et al. (2007), expression of the *ica* operon and biofilm formation are highly variable and biofilm-positive staphylococcal strains (genomic DNA) may not produce a biofilm in some situations but can become biofilm producers in others as a result of changes in environmental factors [27], such as high osmolarity and subinhibitory concentrations of antibiotics. In the study by Kot et al. (2018), the transcription levels were significantly higher in the first hours of biofilm growth compared to planktonic growth, suggesting that these genes are important in the early stage of biofilm growth in which bacterial cells interact with extracellular ligands of the host [28].

The *bhp* gene was only detected in *S. epidermidis* (4.9%), while the *bap* gene was found in 36.4% of all isolates studied, most of them being *S. aureus*. Our findings contrast with Akshatha et al. (2020) who evaluated biofilm formation in *S. aureus* strains obtained from the milk of cows with mastitis and detected the *bap* gene in 12.9% of the isolates [29]. Tormo et al. (2005) observed that *Staphylococcus* strains carrying the *bap* gene were strong biofilm producers even when they did not carry the *icaA*DBC genes [30]. In cases of mastitis, this virulence factor can facilitate the capacity of these bacteria to adhere to the mammary epithelium and to the formation of multiple layers of cells surrounded by the biofilm matrix [31].

Analysis of the clonal profile revealed the formation of eight clusters for *S. aureus*, with the largest cluster (CSA1) comprising isolates from SP and MG. This cluster includes isolates from seven different farms, indicating dissemination of this cluster in the region studied. Among the CoNS, the *S. epidermidis* strains formed four clusters and the *S. chromogenes* strains formed three clusters. Epidemiological studies on *S. aureus* in cattle have shown a large number of molecular profiles to be involved in the etiology of mastitis worldwide, but some profiles tend to predominate in certain geographic regions [32,33].

In the study by Tondo et al. (2000) conducted in Nova Petrópolis, Rio Grande do Sul, PFGE analysis revealed 42 different patterns among 48 *S. aureus* strains isolated from food handlers, raw milk and milk products, demonstrating the diversity of this microorganism in the processing plant [34]. These findings show the lack of an endemic strain among the personnel, although they have worked together for years in the same area. In contrast, we found a large number of isolates that formed clusters with a similarity of 80% or higher, even when the isolates were from different farms and different states.

According to Buzolla et al. (2001) [35], strains with identical genotypes can possess characteristics that confer advantages in terms of their survival in the environment, colonization of the udder and causing disease, such as biofilm expression which was observed in the present study. It can also be indicative of clonal dissemination due to the possible lack of adequate hygiene conditions during milking. Mechanical milking lines are an important source of *Staphylococcus* transmission between dairy herds since these machines can be contaminated with microorganisms originating from the skin of the animal, milk, or the milker’s hands [36].

The prevalent STs in *S. aureus* were ST126 (CC126; 30%) and ST1 (CC1; 30%). MLST has been used to characterize and investigate the distribution of *S. aureus* clones in human infections [37] and in bovine mastitis [38]. However, MLST data for bovine isolates are still sparse.

ST1 (CC1) is widely found in humans, in different animal species, and in cases of bovine mastitis [39,40]. An important association between this clonal complex and infection with *S. aureus* in humans is reported worldwide, including in Brazil. This finding indicates the possible transmission of strains between humans and cattle, although it does not seem to be a frequent event [41].

CC126, together with CC97, has been reported in the literature to be associated with bovine mastitis in different herds and countries. These complexes are rarely isolated from humans [41]. Isolates belonging to CC126 were among those most frequently recovered in this study, a finding showing that different clonal complexes can predominate in different geographic areas. A Brazilian study conducted by Rossi et al. (2019) analyzed and monitored cattle herds with subclinical mastitis over a period of 12 months. The authors demonstrated the persistence of ST126, CC126 in four animals for 4 months [42]. Although few studies have analyzed *S. aureus* isolates in Brazil, all clonal complexes found in the present study were also identified in *S. aureus* strains isolated from mastitis cases in other countries [43].

Regarding the other STs found in our *S. aureus* isolates, we may cite ST8, ST188 and ST746. Boss et al. [44] analyzed 456 bovine strains of *S. aureus* isolated from milk of bovine intramammary infections and bulk tanks obtained from 12 European countries, revealed five major genotypic clusters where one of these clusters with 80 samples presented 83% of ST8 strains [44]. We found ST8 in a sample derived from a cluster with eight isolates typified by PFGE from the state of SC. This finding draws attention because the ST8 belongs to CC8 and according to the studies by Boos et al. [44], animal strains of *S. aureus* evolve from human-adapted strains such that every human strain may be the ancestor of a novel animal- adapted.

For *S. epidermidis*, each cluster exhibited a different ST, with the observation of ST59, ST81, ST48, and ST575, while no exact combination was found for the set of alleles of one isolate. This was a new ST, which was reported to the curator of the MLST site and received the nomenclature ST639. The isolate typed as ST59 and that typed as ST81 are closely related. *Staphylococcus epidermidis* ST59 was previously isolated from bovine mastitis in Germany, from a nasal swab in the Republic of Ireland, and from an animal handler in India (MLST database), demonstrating that this strain can spread to cows and humans. ST81 was isolated from the environment in Poland and from a human wound in Denmark (MLST database).

Taken together, these data reveal the identification of strains with the same evolutionary origin as other isolates around the world, which are known to cause infections in humans and animals, suggesting their ability to spread between these species.

## 4. Materials and Methods

### 4.1. Herds and Sampling

The *Staphylococcus* spp. isolates tested were obtained from a previous longitudinal study that monitored the antimicrobial resistance of udder pathogens in 6 Brazilian states (Rio Grande do Sul (RS), Santa Catarina (SC), Paraná (PR), São Paulo (SP), Minas Gerais (MG), and Pernambuco (PE)) and maintained in the bacterial collection of Empresa Brasileira de Pesquisa Agropecuária (EMBRAPA Gado de Leite, Brazil). In that study, 91 dairy herds were visited four times and the samples were collected on all farms at intervals of 6 months over a period of 2 years. In each sampling, milk samples were collected from all lactating cows of the herd. The total number of collected samples was 28,672. Cows treated with antimicrobial agents were excluded from the sampling. A total of 1365 *S. aureus* and 1484 CoNS were isolated and submitted to antimicrobial susceptibility testing by the disk diffusion method.

### 4.2. Samples Included in the Study

All isolates were submitted to antimicrobial susceptibility testing by the disk diffusion method. After this initial screening conducted by EMBRAPA, 181 *Staphylococcus* spp. that were resistant to oxacillin by this method were selected for the present study.

### 4.3. Bacterial Isolation and Identification

The isolates were identified by biochemical tests and the catalase and coagulase tube tests and biochemical tests (maltose, trehalose, and mannitol) were then carried out to differentiate *S. aureus* from other *Staphylococcus* species [45]. The biochemical tests proposed by Cunha et al. [46] were used for the phenotypic identification of CoNS species and these species were confirmed using the internal transcribed spacer-PCR (ITS-PCR) technique described by Couto et al. (2001) [47].

A total of 181 strains were studied; of these, 82 (45.3%) were identified as *S. aureus* and 99 (54.7%) as CoNS, including 27 (14.9%) *Staphylococcus chromogenes*, 26 (14.4%) *Staphylococcus epidermidis*, 17 (9.4%) *Staphylococcus saprophyticus*, 6 (3.3%) *Staphylococcus warneri*, 6 (3.3%) *Staphylococcus simulans*, 6 (3.3%) *Staphylococcus haemolyticus*, 2 (1.1%) *Staphylococcus xylosus*, 4 (2.2%) *Staphylococcus hominis*, and 5 (2.8%) *Staphylococcus hyicus*.

### 4.4. Detection of the Genes Involved in Biofilm Formation

Genomic DNA was extracted from samples cultured on blood agar, followed by individual inoculation into brain-heart infusion broth and incubation for 24 h at 37 °C. Genomic DNA was extracted using the Illustra^®^ Kit (GE Healthcare, Little Chalfont, Buckinghamshire, UK). PCR for amplification of the genes of the *ica*ADBC operon was performed according to Arciola et al. (2005) [48]. The parameters described by Cucarellas et al. (2004) [18] were used for amplification of the *bap* gene and the method described by Qin et al. (2007) [49] for amplification of the *bhp* gene. This screening was performed to identify which strains carried genes of the operon in their genomic DNA. Next, RT-qPCR using complementary DNA (cDNA) as a template was used to determine whether these genes were expressed.

### 4.5. Expression Analysis of the Genes of the icaADBC Operon by RT-qPCR

The isolates carrying any gene of the *ica*ADBC operon were submitted to RT-qPCR for the analysis of expression and relative quantification of biofilm genes compared to the reference strain.

#### 4.5.1. RNA Extraction and cDNA Synthesis

Total RNA was extracted from samples cultured on blood agar, followed by individual inoculation into brain-heart infusion broth and incubation for 24 h at 37 °C. Total RNA was extracted using the Illustra RNAspin^®^ (GE Healthcare, Amersham, UK) Kit. Possible DNA residues were eliminated by treatment with RNase-free DNAse^®^ (Promega, Madison, WI, USA). The concentration and purity of RNA in the samples were determined in a NanoDrop 2000^®^ spectrophotometer (Thermo Fisher Scientific, Waltham, MA, USA), and A260/A280 and A260/230 ratios, respectively, of approximately 2 were considered adequate for inclusion in the study. Complementary DNA was synthesized with the Superscript VILO^®^ Kit (Life Technologies, Carlsbad, CA, USA) using 1 µg RNA according to manufacturer instructions.

#### 4.5.2. Standardization of the Reaction and Primers

RT-qPCR was performed in a StepOne Plus^®^ thermocycler (Applied Biosystems, Foster City, CA, USA). The reaction mixture contained 10 μL SYBR Green PCR Master Mix, 1.5 μL cDNA, 0.5 pMol/µL forward and reverse primers, and nuclease-free water in a total volume of 20 μL. The reactions were run in duplicate for each target and the level of gene expression was calculated based on the threshold cycle (CT), in which the gene encoding the 16S rRNA was used as normalizer (endogenous control) and the genes of the *ica* operon (*ica*ADBC) as calibrators. The reaction for standardization of the primers showed amplification of a single product for all genes tested in the absence of contamination or background noise. Melting curves were constructed for each gene studied and each curve had only one peak where the variation in temperature was not greater than 0.5 °C per sample in each group of genes analyzed. The primers described by Vandecasteele et al. (2003) [50] were used for the *ica*A gene, those described by Klug et al. (2003) [51] for *icaB*, and the primers described by Tan et al. (2012) [52] for *ica*C and *ica*D.

#### 4.5.3. Relative Standard Curve

The data of the cDNA curves for each target were obtained in reactions performed in triplicate and serially diluted 1:10, resulting in 150, 15, 1.5, 0.15 and 0.015 ng per reaction. The following parameters were considered: variation in CT less than 0.5 between triplicates, R2 > 0.9, efficiency of 90 to 105%, and a slope of 3.32. The threshold of each primer pair was established from the standard curves.

#### 4.5.4. Analysis of Gene Expression in the Isolates

The cDNA of all isolates carrying genes of the *ica* operon detected by conventional PCR in genomic DNA was submitted to RT-qPCR for detection of the 16SrRNA gene used as an endogenous control. Once the positivity for the 16S rRNA gene was confirmed, the expression of the genes was also analyzed. Relative quantification of biofilm gene expression by each isolate was compared to the expression of reference strains (ATCC 35985: biofilm producer and ATCC 1222: non-biofilm producer). The ΔΔCT value was calculated for relative quantification [53].

### 4.6. Determination of Oxacillin and Vancomycin Inhibitory Concentrations by the E-Test

In vitro susceptibility of the isolates to oxacillin and vancomycin was tested. The minimum inhibitory concentration (MIC) of these drugs was determined by the E-test. Inocula adjusted to a turbidity corresponding to a 0.5 McFarland standard were inoculated into Mueller-Hinton medium and incubated at 35 °C. The results were read at the intersection of the MIC scale on the strip and the ellipse-shaped bacterial growth inhibition zone. An international reference strain (*S. aureus* ATCC 29213) was included as a control.

### 4.7. Pulsed-Field Gel Electrophoresis

PFGE of the *Staphylococcus* spp. isolates was performed according to the modified protocol of McDougal et al. (2003) [24]. For similarity analysis, the Dice correlation coefficient was calculated and a dendrogram was generated by the UPGMA method (unweighted pair group method using arithmetic averages) using the BioNumerics^®^ software (version 7.0; Applied Maths, Sint-Martens-Latem, Belgium, 2015). A similarity coefficient of 80% was chosen for the definition of the clusters.

### 4.8. Geographic Distribution of Staphylococcus *spp.* Clones

For the classification of the PFGE clusters, the strains were analyzed regarding their location and distribution across the different states included in the study (PR, SC, SP, RS, MG, and PE). The geographic coordinates of each farm were used, with the milking room serving as a reference. These data were extracted from the questionnaires applied in previous epidemiological studies conducted on these farms. The image was generated with the Arcgis program. The farms were georeferenced and imported to the software using the Geographic Coordinates System GCS Sirgas 2000 and Datum planimetric Sirgas 2000.

### 4.9. Multilocus Sequence Typing

MLST was performed as described by Enright et al. (2000) [54]. After amplification and sequencing of the seven housekeeping genes, the sequences were analyzed with the BioNumerics^®^ software (version 7.0; Applied Maths, Sint-Martens-Latem, Belgium, 2015) and compared with the online database (http://www.mlst.net) to obtain the identification number of each allele. The combination of these alleles indicates the ST to which the isolates belong.

## 5. Conclusions

The determination of the resistance of *S. aureus* and CoNS to oxacillin by the E-test demonstrated that CoNS had higher resistance rates. When analyzing resistance to vancomycin, all samples were sensitive. The presence and expression of the genes encoding the biofilm were more present in *S. aureus*, with the most expressed genes being *ica*A and *ica*D. Molecular typing by PFGE revealed a large cluster of *S. aureus* that includes isolates from different properties and locations. In other species it was also possible to verify the formation of clusters with these characteristics. With respect to MLST, ST126 and ST1 were the prevalent sequence types in *S. aureus*, while in *S. epidermidis* all sequence types were different, with a new ST, which received the nomenclature ST639. The identification of strains with the same evolutionary origin as other isolates around the world, which are known to cause infections in humans and animals, suggesting their ability to spread between these species. The present study highlights the need to evaluate the microbiological and molecular characteristics of bovine mastitis isolates so that effective infection control measures.

## Figures and Tables

**Table 1:** Distribution of detection (gDNA) and expression (cDNA) of genes involved in biofilm formation according to *Staphylococcus* species.

Species (N)	gDNA ^1^ *ica*A	cDNA ^2^ *ica*A	gDNA *ica*B	cDNA *ica*B	gDNA *ica*C	cDNA *ica*C	gDNA *ica*D	cDNA *ica*D
*S. aureus* (82)	71	58	4	4	26	18	69	62
*S. chromogenes* (27)	0	0	4	1	6	1	12	4
*S. epidermidis* (26)	5	1	8	0	12	3	17	3
*S. saprophyticus* (17)	2	0	4	0	2	0	8	0
*S. haemolyticus* (6)	1	1	0	0	4	2	4	3
*S. simulans* (6)	0	0	1	1	3	0	5	0
*S. warneri* (6)	0	0	3	0	1	0	4	5
*S. hyicus* (5)	0	0	0	0	2	0	4	1
*S. hominis* (4)	0	0	0	0	0	0	2	0
*S. xylosus* (2)	0	0	0	0	1	0	2	2
Total (181)	79	60	24	6	57	24	127	80

^1^ genomic DNA; ^2^ co mplementary DNA.

**Table 2:** Interpretation of susceptibility to oxacillin and vancomycin by the E-test.

Antimicrobial						
	Oxacillin			Vancomycin		
**Species**	**MIC_50_**	**MIC_90_**	**Resistant**	**MIC_50_**	**MIC_90_**	**Resistant**
***S. aureus***	0.094	0.25	1	0.50	1.0	0
**CoNS**	0.25	1.5	32	1.0	1.5	0

Oxacillin breakpoint: *Staphylococcus aureus*: susceptible ≤2; resistant ≥4 µg/mL. CoNS (coagulase-negative *Staphylococcus*): susceptible ≤0.25; resistant ≥0.5 µg/mL. Vancomycin breakpoint: *Staphylococcus* spp.: susceptible <4 [23]. MIC, minimum inhibitory concentration.

**Table 3:** Electrophoretic pattern and expression of genes of the *ica*ADBC operon in *Staphylococcus* spp. clones isolated from bovine subclinical mastitis cases in different Brazilian states.

Species	Cluster	No. of Isolates	Expression of *ica*ADBC Genes	Origin
*S. aureus*	CSA1	31	*ica*A (23); *ica*B (0); *ica*C (4); *ica*D (23)	SP; MG
	CSA2	3	*ica*A (1); *ica*B (0); *ica*C (0); *ica*D (1)	SP; RS
	CSA3	7	*ica*A (5); *ica*B (2); *ica*C (5); *ica*D (7)	PR
	CSA4	5	*ica*A (3); *ica*B (0); *ica*C (2); *ica*D (4)	PR; SC; SP
	CSA5	6	*ica*A (5); *ica*B (0); *ica*C (1); *ica*D (5)	SC
	CSA6	5	*ica*A (4); *ica*B (0); *ica*C (2); *ica*D (5)	SC
	CSA7	3	*ica*A (2); *ica*B (0); *ica*C (2); *ica*D (2)	RS; SC; SP
	CSA8	4	*ica*A (3); *ica*B (1); *ica*C (0); *ica*D (3)	SC
*S. chromogenes*	CSC1	11	*ica*A (0); *ica*B (1); *ica*C (0); *ica*D (2)	SC; SP
	CSC2	4	*ica*A (0); *ica*B (0); *ica*C (0); *ica*D (0)	SC; SP
	CSC3	6	*ica*A (0); *ica*B (0); *ica*C (0); *ica*D (2)	PR; SC; SP
*S. epidermidis*	CSE1	4	*ica*A (0); *ica*B (0); *ica*C (0); *ica*D (1)	PR; SC
	CSE2	3	*ica*A (0); *ica*B (0); *ica*C (1); *ica*D (1)	MG; SC; SP
	CSE3	3	*ica*A (0); *ica*B (0); *ica*C (0); *ica*D (0)	SC
	CSE4	3	*ica*A (1); *ica*B (0); *ica*C (1); *ica*D (1)	PR
*S. saprophyticus*	CSS1	3	*ica*A (0); *ica*B (0); *ica*C (0); *ica*D (0)	SP
*S. simulans*	CSI1	3	*ica*A (0); *ica*B (1); *ica*C (0); *ica*D (0)	RS; SC

CSA: cluster *Staphylococcus aureus*; CSC: cluster *Staphylococcus chromogenes*; CSE: cluster *Staphylococcus epidermidis*; CSS: cluster *Staphylococcus saprophyticus*; CSS: Cluster *Staphylococcus simulans*. SP: São Paulo; MG: Minas Gerais; RS: Rio Grande do Sul; PR: Paraná; SC: Santa Catarina.

**Table 4:** Identification of the sequence type (ST) of *Staphylococcus aureus* isolates.

Origin	PFGE Cluster	Isolates	Alleles							ST
			*arcC*	*aroE*	*glpF*	*gmk*	*pta*	*tpi*	*yqiL*	
**MG**	CSA1	2	3	68	1	4	1	5	40	126
**SP**	CSA1	91	3	68	1	4	1	5	40	126
**SP**	CSA2	88	3	68	1	4	1	5	40	126
**PR**	CSA3	18	1	1	1	1	1	1	1	1
**PR**	CSA3	178	1	1	1	1	1	1	1	1
**SC**	CSA4	40	1	1	1	1	1	1	1	1
**SC**	CSA5	54	3	1	1	8	1	1	1	188
**SC**	CSA6	43	3	1	1	1	1	5	92	746
**RS**	CSA6	170	3	1	1	1	1	5	92	746
**PR**	CSA7	24	1	1	1	1	1	1	1	1
**SC**	CSA8	71	3	3	1	1	4	4	3	8

PFGE: Pulsed Field Gel Electrophoresis; MG: Minas Gerais; SP: São Paulo; PR: Paraná; SC: Santa Catarina, RS: Rio Grande do Sul; CSA: Cluster *Staphylcoccus aureus*.

**Table 5:** Identification of the sequence type (ST) of *Staphylococcus epidermidis* isolates.

Origin	PFGE Cluster	Isolates	Alleles							ST
			*arcC*	*aroE*	*gtr*	*mut*	*pyr*	*tpi*	*yqiL*	
**PR**	CSE1	16	1	1	1	6	2	1	7	575
**SC**	CSE1	75	2	17	1	1	2	1	1	81
**MG**	CSE2	1	2	1	1	1	2	1	1	59
**SC**	CSE3	81	7	1	2	6	2	1	1	639
**PR**	CSE4	11	7	1	2	2	4	1	4	48

CSE: cluster Staphylococcus epidermidis.

**Table 6:** Susceptibility profile of the isolates according to sequence type (ST).

Species	Strain	ST	Oxacillin	Vancomycin
*S. epidermidis*	1	59	R	S
*S. aureus*	2	126	S	S
*S. epidermidis*	11	48	R	S
*S. epidermidis*	16	575	S	S
*S. aureus*	18	1	S	S
*S. aureus*	24	1	S	S
*S. aureus*	43	746	S	S
*S. aureus*	54	188	S	S
*S. aureus*	71	8	S	S
*S. epidermidis*	75	81	R	S
*S. epidermidis*	81	639	S	S
*S. aureus*	88	126	S	S
*S. aureus*	91	126	S	S
*S. aureus*	170	746	S	S
*S. aureus*	178	1	S	S

R: resistant; S: susceptible.

## Supplementary Materials

The following are available online at https://www.mdpi.com/2079-6382/9/12/888/s1, Table S1: Brazilian state of origin (Minas Gerais−MG; Pernambuco−PE; Paraná−PR; Rio Grande do Sul−RS; Santa Catarina−SC), identification number of the farm, geographic coordinates, number of the isolate, cluster, and sequence type (ST).
