# Supplementary material for: Staphylococcus spp. Isolated from Bovine Subclinical Mastitis in Different Regions of Brazil: Molecular Typing and Biofilm Gene Expression Analysis by RT-qPCR
Source: Antibiotics (Basel). 2020 Dec 10;9(12):888. doi: 10.3390/antibiotics9120888 (PMC7764751; doi:10.3390/antibiotics9120888)
Supplement: Supplementary file 1 [file antibiotics-09-00888-s001.pdf]

Table S1. Brazilian state of origin (Minas Gerais – MG; Pernambuco - PE; Paraná – PR; Rio Grande do Sul – RS; Santa Catarina – SC), identification number of the farm, geographic coordinates, number of the isolate, cluster, and sequence type (ST).

State	Number of farm	Latitude	Longitude	Isolate	Cluster	ST
MG	1	-29.37184	-51.87331	1	CSE4	59
MG	2	-29.37184	-52.07478	2	CSA1	126
MG	1	-29.37184	-52.07478	3		
MG	1	-29.37184	-52.07478	4		
PR	16	-9.16839	-36.68521	5		
PR	12	-8.65609	-36.32987	6	CSC1	
PR	14	-9.08953	-36.63096	7		
PR	14	-26.05670	-53.36940	8		
PR	10	-27.00000	-53.00000	9	CSE2	
PR	13	-22.00000	-44.00000	10	CSE2	
PR	17	-22.08636	-43.82967	11	CSE2	48
PR	11	-22.00000	-44.00000	12	CSA2	
PR	18	-22.00000	-44.00000	13	CSA2	1
PR	15	-25.99030	-53.65860	14	CSE3	
PR	9	-25.79830	-53.56970	15	CSA2	
PR	15	-25.56750	-53.84330	16	CSE3	575
PR	13	-25.56750	-53.84330	17		
PR	6	-25.67544	-53.54374	18	CSA2	
PR	8	-25.66894	-53.81517	19		
PR	11	-25.96030	-53.69420	20		
PR	18	-25.72953	-53.71555	21	CSE3	
PR	9	-26.05940	-53.36000	22	CSA2	
PR	6	-26.05670	-53.36940	23		
PR	7	-25.84890	-53.77420	24	CSA5	1

PR	7	-26.05670	-53.36940	25	CSA5	
PR	12	-25.66894	-53.81517	26		
PR	12	-25.92110	-53.50500	27	CSA5	
PR	10	-25.72953	-53.54374	28	CSA2	
PR	9	-25.72953	-53.71555	29	CSA2	
PR	9	26.05940	-53.36000	30		
SC	34	-25.84890	-53.77420	31		
SC	35	-25.92110	-53.50500	32	CSA4	
SC	27	-25.96030	-53.69420	33	CSA4	
SC	31	-25.96030	-53.69420	34	CSA3	
SC	31	-25.79830	-53.56970	35	CSA3	
SC	29	-25.79830	-53.56970	36	CSA3	
SC	29	-25.67544	-53.54374	37	CSA3	
SC	31	-25.84890	-53.77420	38	CSA4	
SC	29	-25.84890	-53.77420	39	CSA3	
SC	25	-53.00000	-27.00000	40	CSA2	1
SC	26	-26.87511	-52.40419	41	CSA6	
SC	25	-53.00000	-27.00000	42	CSA2	
SC	35	-27.00000	-53.00000	43	CSA4	746
SC	35	-27.00000	-53.00000	44		
SC	25	-26.72670	-52.72446	45		
SC	32	-26.72670	-52.72446	46		
SC	30	-27.00000	-53.00000	47		
SC	30	-26.72670	-52.72446	48	CSC3	
SC	30	-26.95589	-52.53599	49		
SC	26	-27.00000	-53.00000	50	CSC2	
SC	26	-26.95589	-52.53599	51	CSE1	
SC	33	-26.87511	-52.40419	52		

SC	33	-26.87511	-52.40419	53		
SC	33	-26.95589	-52.53599	54	CSA3	188
SC	33	-27.00000	-53.00000	55	CSC2	
SC	33	-26.58749	-53.58534	56	CSA6	
SC	34	-26.58749	-53.58534	57		
SC	25	-26.58749	-53.58534	58		
SC	25	-27.00000	-53.00000	59		
SC	25	-27.00000	-53.00000	60		
SC	28	-26.72670	-52.72446	61		
SC	32	-26.72670	-52.72446	62		
SC	32	-26.72670	-52.72446	63		
SC	26	-26.72670	-52.72446	64		
SC	35	-26.72670	-52.72446	65	CSC1	
SC	32	-53.00000	-27.00000	66	CSC1	
SC	30	-26.95589	-52.53599	67	CSA6	
SC	31	-26.95589	-52.53599	68		
SC	31	-26.95589	-52.53599	69		
SC	33	-26.85790	-52.56528	70	CSC1	
SC	34	-27.00000	-53.00000	71	CSA6	8
SC	30	-27.00000	-53.00000	72	CSC3	
SC	30	-27.00000	-53.00000	73		
SC	26	-26.87511	-52.40419	74	CSC2	
SC	31	-27.00000	-53.00000	75	CSE3	81
SC	31	-27.00000	-54.00000	76	CSC2	
SC	31	-27.00000	-53.00000	77		
SC	33	-27.00000	-53.00000	78	CSE1	
SC	33	-26.72670	-52.72446	79	CSA2	
SC	33	-53.00000	-27.00000	80	CSC2	

SC	34	-27.00000	-54.00000	81	CSE1	639
SC	32	-27.00000	-54.00000	83		
SC	32	-27.00000	-53.00000	85	CSE4	
SC	32	-27.00000	-53.00000	86		
SC	33	-27.00000	-53.00000	87		
SP	39	-27.00000	-53.00000	88	CSA1	126
SP	39	-26.72670	-52.72446	89	CSA1	
SP	39	-26.72670	-52.72446	90	CSA1	
SP	39	-26.72670	-52.72446	91	CSA1	126
SP	39	-53.00000	-27.00000	92	CSA1	
SP	39	-27.00000	-53.00000	93	CSA1	
SP	39	-27.00000	-53.00000	94	CSA1	
SP	46	-27.00000	-53.00000	96		
SP	41	-26.72670	-52.72446	98	CSS1	
SP	41	-22.10793	-4719743	99	CSS1	
SP	44	-22.10793	-4719743	100	CSC3	
SP	44	-22.10793	-4719743	102	CSC2	
SP	38	-22.10793	-4719743	103		
SP	38	-22.10793	-4719743	105	CSS1	
SP	47	-22.10793	-4719743	106		
SP	47	-22.10793	-47.19743	107		
SP	47	-21.57330	-47.27147	108	CSC2	
SP	47	-22.03783	-47.31533	109	CSC2	
SP	47	-22.03783	-47.31533	110		
SP	44	-22.08642	-47.30266	111	CSA1	
SP	44	-22.08642	-47.30266	112		
SP	42	-22.02294	-47.21954	113		
SP	45	-22.02294	-47.21954	114	CSA4	

SP	36	-22.03558	-47.21020	115	CSA1	
SP	36	-22.03558	-47.21020	116	CSA1	
SP	36	-22.03558	-47.21020	117	CSA1	
SP	46	-22.03558	-47.21020	118		
SP	40	-22.03558	-47.21020	119		
SP	40	-22.08642	-47.30266	121		
SP	36	-22.08642	-47.30266	122	CSA1	
SP	36	-21.59518	-47.36216	123	CSA1	
SP	36	-22.11392	-47.27687	124	CSA1	
SP	36	-21.47006	-47.19743	125	CSA1	
SP	36	-21.47006	-47.19743	126	CSA1	
SP	43	-21.47006	-47.19743	127	CSA1	
SP	43	-21.57330	-47.27147	128	CSA1	
SP	43	-22.01764	-47.35078	129	CSA1	
SP	43	-22.01764	-47.35078	130	CSA1	
SP	43	-21.47006	-47.19743	131	CSA1	
SP	39	-21.47006	-47.19743	132		
SP	36	-21.47006	-47.19743	133	CSA1	
SP	36	-21.47006	-47.19743	134	CSA1	
SP	36	-21.47006	-47.19743	135	CSA1	
SP	44	-21.99696	-47.42619	136	CSA1	
SP	47	-21.99696	-47.42619	137	CSA2	
SP	44	-21.99696	-47.42619	138		
SP	48	-21.99696	-47.42619	139		
SP	44	-21.99696	-47.42619	140		
SP	44	-22.10793	-47.19743	141		
SP	46	-21.47006	-47.19743	142	CSC2	
SP	46	-21.47006	-47.19743	143	CSC1	

SP	46	-21.47006	-47.19743	144	CSC2	
SP	47	-22.08642	-47.30266	146		
SP	36	-22.03558	-47.21020	147	CSA1	
SP	36	-22.08642	-47.30266	148	CSA1	
SP	37	-21.52129	-47.40640	149	CSA1	
SP	43	-22.08642	-47.30266	150	CSA1	
SP	43	-22.08642	-47.30266	151	CSA1	
SP	43	-21.57330	-47.27147	152	CSA1	
SP	46	-21.57330	-47.27147	153	CSC2	
SP	44	-21.57330	-47.27147	154		
SP	36	-22.03558	-47.21020	155	CSC1	
SP	43	-21.47006	-47.19743	156	CSA1	
SP	43	-21.47006	-47.19743	157	CSC3	
SP	43	-21.97077	-46.79473	159		
SP	43	-21.99606	-47.42619	160		
SP	43	-21.99606	-47.42619	161		
SP	46	-21.99606	-47.42619	162		
SP	46	-21.57330	-47.27147	163		
RS	19	-22.08642	-47.30266	164		
RS	22	-21.47006	-47.19743	165		
RS	24	-21.99606	-47.42619	166	CSA4	
RS	24	-21.99606	-47.42619	167		
RS	20	-21.99606	-47.42619	168		
RS	20	-21.99606	-47.42619	169	CSA4	
RS	23	-21.99606	-47.42619	170	CSA4	746
RS	23	-21.57330	-47.27147	171		
RS	21	-21.57330	-47.27147	172		
RS	21	-29.21467	-52.02655	173		

RS	21	-29.21467	-52.02655	174		
PE	3	-29.23571	-51.86962	175		
PE	4	-29.23571	-51.86962	176		
PE	5	-28.96995	-52.01067	177		
PR	15	-28.96995	-52.01067	178	CSA2	1
SC	32	-29.37184	-51.87331	181		

CSA: cluster *Staphylococcus aureus*; CSC: cluster *Staphylococcus chromogenes*; CSE: cluster *Staphylococcus epidermidis*; CSS: cluster *Staphylococcus saprophyticus*; CSI: Cluster *Staphylococcus simulans*.
